# Co-expression of caspase-3 or caspase-8 with galanin in the human stomach section affected by carcinoma

**DOI:** 10.1007/s10495-018-1470-y

**Published:** 2018-07-17

**Authors:** Anna Kozłowska, Piotr Kozera, Mariusz Majewski, Janusz Godlewski

**Affiliations:** 1 Department of Human Physiology, School of Medicine, Collegium Medicum, University of Warmia and Mazury in Olsztyn, Warszawska Av 30, 10-082 Olsztyn, Poland; 2 Department of Human Histology and Embryology, School of Medicine, Collegium Medicum, University of Warmia and Mazury in Olsztyn, Warszawska Av 30, 10-082 Olsztyn, Poland

**Keywords:** Caspase-3, Caspase-8, Galanin, Gastric cancer

## Abstract

Neoplastic process may cause distinct changes in the morphology, i.e. size and number of the neurons of the neuronal plexuses forming the enteric nervous system (ENS) of the human intestine. Moreover, it was also reported that these changes were not directly associated with apoptosis. Thus, the main aim of this study was to determine the atrophic changes of myenteric plexuses (MPs) in the vicinity of cancer invasion and the potential reason which may be responsible for these changes if they occur. Tissue samples from the stomach were collected from ten patients which undergo organ resection due to cancer diagnosis. Samples were taken from the margin of cancer invasion and from a macroscopically-unchanged part of the stomach wall. Triple-immunofluorescence staining of the 10-µm-thick cryostat sections was used to visualize the co-expression of caspase-3 (CASP3) or caspase-8 (CASP8) with galanin (GAL) in the MPs of ENS. Microscopic observations of MPs located closely to gastric cancer invasion showed that they were significantly smaller than plexuses located distally. The percentage of neurons containing CASP3 within MPs located close to cancer-affected regions of the stomach was higher, while containing CASP8 was lower compared to the unchanged regions. Additionally, elevated high expression of CASP3 or CASP8 in the neurons from MPs was accompanied by a decreased expression of GAL. To our knowledge, this is the first report describing the decomposition of MPs within cancer-affected human stomach wall and the possible role of apoptosis in this process.

## Introduction

Enteric nervous system (ENS) is the most complex and the largest division of the autonomic nervous system, estimated to be composed of approximately 500 million of neurons [1]. It should, however, be emphasized that the enteric ganglia, forming the myenteric (MPs) as well as the submucosal plexuses (SPs) of the ENS are composed not only of enteric neurons, but contain also numerous glia cells. Furthermore, as may be judged from numerous studies, both ganglionic components cooperate closely with the interstitial cells of Cajal, regulating together gastrointestinal functions [2–4]. From the histological point of view, ENS neurons are accumulated into either MPs or SPs (Auerbach’s plexus, Meissner’s plexus, respectively). From the physiological point of view, MPs, located between longitudinal and circular muscles of *muscularis externa* is responsible for control of peristalsis, while neurons forming SPs are responsible for intestinal absorption and secretion [1].

Latest studies showed that ENS may undergo severe decomposition under various pathological conditions. For example, it was reported that MPs in the vicinity of the colorectal cancer (CRC) tissue were significantly smaller and had lower number of neurons per plexus compared to distally located plexuses [5, 6]. Similar observations were also reported in the colon wall of patients with diabetes [7] and severe constipation [8]. One of the reasons of described above changes in ENS may be apoptosis that is known to be a cause of death in some cells (e.g. neurons [9, 10]). It is generally known that this process leads to dismantling the intracellular components while avoiding inflammation and it can be triggered by an internal or external stimulus [11]. These stimuli are transmitted by caspases (CASP) which can be divided into initiator (CASP8; CASP9) and executioner endoproteases (CASP3; CASP6; CASP7). Caspases can be activated via extrinsic and intrinsic pathways and both coincide at a common point, which is activation of executioner caspase that leads to protein proteolysis and DNA damage [5, 11]. Galanin (GAL) can be another candidate which may play important role in this process. GAL is commonly known to take part in the regulation of inflammatory processes and neuronal plasticity, acting as growth factors for neurons [12, 13]. In the ENS, this neuropeptide is hypothesized to influence the gastrointestinal motility (by inhibition of acetylcholine and substance P release from excitatory motor neurons) as well as secretion (through GAL 1 receptors—GAL-R_1_) [14]. Elevated GAL levels in the serum and colon tissues were observed in patients with CRC [6]. Thus it has been proposed that an increase in number of GAL-immunoreactive (-IR)  neurons can be associated with adaptive and neuroprotective possibilities of the enteric neurons that is well-documented in many experimental models of brain and neurons injury [15, 16].

Thus, the above-mentioned data strongly suggest that the invasion by neoplastic cells may lead to profound changes in the morphology, composition, chemical coding as well as functionality of MPs also in human stomach. However, due to severe paucity of data dealing with this aspect of gastro-intestinal tumorigenesis, the goal of the present study was restricted to compare (1) changes in the morphology of the MPs (focusing on the size of particular ganglia and the number of neurons forming them) and (2) the dynamic of apoptotic process in the population of neurons formed MPs (focusing on the co-expression patterns of caspase-3 (CASP3) and caspase-8 (CASP8) with galanin (GAL; implicated to play, among others, also an important neuroprotective function) in both the cancer-affected as well as microscopically cancer-free regions of the human stomach wall.

## Materials and methods

### Patient recruitment and specimen collection

The post-operative material used in the present study was obtained, during surgery, at the Department of Oncological Surgery of the Regional Oncological Centre in Olsztyn, Poland. The tissue samples were collected from ten patients with stomach cancer diagnosis. The study group consisted of 3 women and 7 men, the mean age of the patients was 67.0 ± 11.9 years (range: 51–85 years). Post-operative pathomorphological analyses confirmed that patients included in this study formed a homogenous group with the same degree of adenocarcinoma invasion within the stomach wall, defined as T_3_ on the TNM scale by the American Joint Committee on Cancer (AJCC) staging.

The samples from the present study were collected based on a protocol approved by the Bioethics Commission (No. 18/2012) of University of Warmia and Mazury in Olsztyn and written informed consent was obtained from all patients in the study. Moreover, all patients did not have a second serious illness or neo-adjuvant chemo- or/and radiotherapy.

Directly after surgical organ resection, small samples (10 mm × 10 mm × 10 mm) were obtained in duplicate from the same gross-anatomical localization (body of the stomach) in each patient enrolled in the study. First sample comprised the margin of cancer invasion, the second one was taken from macroscopically-unchanged stomach wall, at a distance of 5–8 cm from the tumor (surgical margin; if neoplastic cells were not observed during histopathological examination, the sample was used as a control tissue). In the next step, the samples were fixed by immersion in the 4% buffered paraformaldehyde (pH 7.4) for 120 min, washed twice in 0.1 M phosphate buffer (pH 7.4, 4 °C) over 3 days and then stored in 18% buffered sucrose solution containing 0.01% sodium azide (pH 7.4) for 7 days at 4 °C until freezing and sectioning.

### Immunofluorescence procedures

Prior to sectioning, each sample studied was mounted on a small pad formed from frozen Tissue-Tek OCT compound (Sakura Finetek, USA). Next the 10 mm-thick cryostat (HM525 Zeiss, Germany) sections of stomach wall were subjected to a triple-immunofluorescence staining technique, using antibodies listed in Table 1. In brief, frozen sections were always air-dried at room temperature (RT) for 45 min, rinsed three times in phosphate-buffered saline (PBS, pH 7.4), and then incubated for 1 h with a blocking solution (BS) containing 1% Triton X-100, 0.1% bovine serum albumin, 0.05% thimerosal (all from Sigma-Aldrich, St. Louis, MO), 0.01% NaN_3_ (POCH, Gliwice, Poland) and 10% normal goat serum (Jackson Immunoresearch, West Grove, PA, USA) in 0.01 M PBS (pH 7.4) to reduce non-specific background staining. This solution was also used as a diluent of primary and secondary antibodies.

**Table 1:** Immunoreagents used

Antisera	Code	Host species	Dilution	Supplier
Primary antibody				
Active caspase-3	ab2302	Rabbit	1:1500	Abcam, Cambridge, UK
Caspase-8	ab25901	Rabbit	1:1000	Abcam, Cambridge, UK
Galanin	T-5034 (GHC 7100)	Guinea pig	1:1200	Bachem AG, Bubendorf, CH
PGP 9.5	7863-2004	Mouse	1:950	Biogenesis, Kingstone, NH, USA
Secondary antibody				
Biotinylated policlonal anti-rabbit	E0432	Goat	1:1000	Dako, Glostrup, DK
Fluorescein-conjugated Affini Pure anti-guinea pig	706-096-148	Donkey	1:450	Jackson Immunoresearch, West Grove, PA, USA
AMCA-Affini Pure anti-mouse	715-156-151	Donkey	1:75	Jackson Immunoresearch,West Grove, PA, USA
Cy^TM^3-conjugated streptavidin	016-160-084		1:4500	Jackson Immunoresearch, West Grove, PA, USA

For the triple-labelling immunofluorescence, a mixture of three primary antibodies raised in different host species was used. After the incubation with primary antibodies, sections were rinsed in PBS (3 × 15 min) and incubated for 1 h with a mixture of appropriate AMCA- and FITC-conjugated secondary antisera and biotinylated donkey anti-rabbit antibody. The latter antibody was finally visualized by additional incubation of sections with streptavidin-CY3 complex for 1 h. Following subsequent rinsing in PBS (3 × 15 min), sections were coverslipped with carbonate-buffered glycerol (pH 8.6).

### Mayer’s hematoxylin staining

Cryostat sections of the cancer-affected and cancer-free stomach wall were stained for 5 min with Mayer’s hematoxylin (Sigma-Aldrich, St. Louis, MO). After careful washing, stained sections were cover slipped, analyzed and photographed using a light microscope (BX-41, Olympus, Tokyo, Japan) and XC-50 camera (Olympus) under 200× magnification.

### Specificity test of labelling procedures

The specificity of primary antisera was tested as follows: sections were incubated with an antibody that had been pre-absorbed with synthetic antigen (10 µg of antigen per 1 ml of diluted antiserum); the primary antibody was omitted from the incubation; or normal rabbit, guinea pig or mouse serum was substituted for the primary antibody.

### Morphological analyses

After being photographed, 100 selected randomly MPs (n = 50—from cancer-affected wall; n = 50—samples of morphologically unchanged wall of stomach as control tissues) were measured using Cell Sens Dimension software and expressed in µm^2^. Moreover, the number of all PGP 9.5-immunoreactive (-IR) neurons in those plexuses was counted manually and expressed as mean per plexus.

### Counting of neurons

Triple-immunolabelled neurons were analyzed under an Olympus BX61 microscope (Olympus, Tokyo, Japan) equipped with the epi-fluorescence kit and appropriate filter sets for AMCA (V1 module, excitation range 330–385 nm and barrier filter at 420 nm), FITC (B1 module, excitation filter 450–480 nm) and CY3 (G1, excitation filter 510–550 nm). Microphotographs were acquired using a 20× UPlanSApo objective (0.75 NA) and a PC equipped with a CCD camera operated by Cell Sens Dimension image analyzing software (Olympus, Poland). The slides were photographed with a confocal laser microscope (LSM 700, Zeiss). For each patient, twelve cryostat sections (10 µm thick) obtained from the two parts of the stomach wall (n = 6—samples from cancer-affected wall; n = 6—samples as control) was labeled and then analyzed. A distance between sections of 150 µm avoided double counting the same neuron within MPs in adjacent sections. In each patient the number of neurons containing PGP 9.5 (used as pan-neuronal marker, [17]) and co-expressing CASP3 and/or GAL as well as CASP8 and/or GAL was counted in each of twelve cryostat sections. All counts were made on coded slides prepared by the first author. To avoid fluorescence fading, a test frame was digitally recorded before counting. Such digital frames were in the form of stacks, which consisted of three microphotographs representing red, green and blue immunofluorescence channels. Saved stacks were then evaluated by two independent experimenters, being blind to the parameters of the studied tissue. The results of these counts showed high inter-rated reliability (Pearson R = 0.82, P < 0.01).

### Statistical analysis

Data concern the differences in the area of MPs and number of neurons inside them as well as of differences in the immunoreactivity to CASP3 or CASP8 with GAL found between the cancer-affected and the control part of stomach wall were analyzed using the Mann–Whitney U-test. In all performed analyses, the results were considered as statistically significant (p < 0.05).

## Results

### Morphometry of myenteric plexuses (MPs) in the vicinity of and distally from gastric cancer invasion

The morphological analysis of MPs has shown notable atrophy in the vicinity of gastric cancer invasion. The atrophy is characterized by smaller mean plexus area when compared to control plexuses located distally from the cancer invasion. No differences between cancer-affected and cancer-free tissue in the mean number of neurons per plexus were observed. The mean and total (± SEM) number of neurons in MPs as well as mean plexus areas are shown in Tables 2 and 3.

**Table 2:** Mean area and number of neurons in the stomach myenteric plexuses located close to and distally from cancer invasion. Data were pooled and presented as the mean (range) of ten patients

Mean plexus area [µm^2^]			Mean number of neurons per plexus		
Close to tumor invasion	Distally from tumor	p	Close to tumor invasion	Distally from tumor	p
Myenteric plexus					
31.464 ± 2723	52.185 ± 5901	0.003	3.55 ± 0.36	5.13 ± 0.68	ns

*ns* no statistical differences were observed

**Table 3:** The total number of neurons in the stomach myenteric plexuses located close to and distally from cancer invasion. Data were pooled and presented as the mean (range) of ten patients

The total number of PGP 9.5-labeled neurons for combination CASP3 with GAL in the human stomach wall	1165 [100%]	The total number of PGP 9.5-labeled neurons for combination CASP8 with GAL in the human stomach wall	1260 [100%]
Cancer-affected	512 [43.95%]	Cancer-affected	528 [41.90%]
Cancer-free	653 [56.05%]	Cancer-free	732 [58.10%]

### Mayer’s hematoxylin staining

The conventional H&E staining did not reveal any cancer invasion-related changes in specimens used as “control” tissues.

### The analysis of the co-localisation of CASP3 or CASP8 with galanin in the neurons of myenteric plexuses (MPs) in the vicinity of and distally from gastric cancer invasion

#### Caspase-3 immunoexpression

**Fig. 1:**
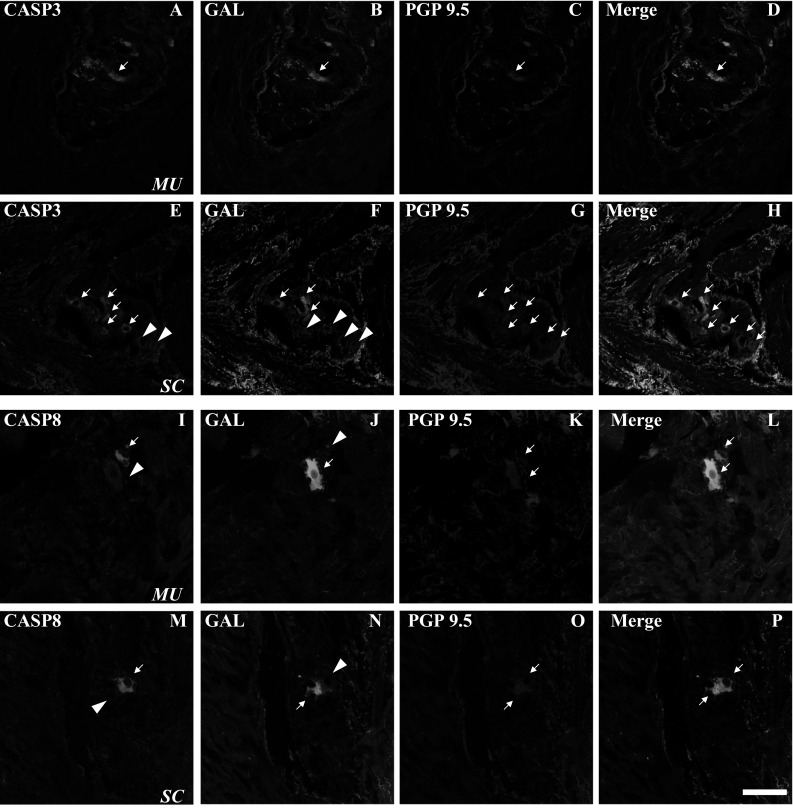
Representative images of neurons located in myenteric plexuses (MPs) of human stomach wall: macroscopically-unchanged (MU; **a**–**d, i**–**l**) and cancer-affected (SC; **e**–**h, m**–**p**). Small-sized arrows show single-, double- or triple-stained cells, while large-sized arrows pointed out the lack of co-expression. All the images are taken separately from red (positive for: CASP3—**a, e** or CASP8—**i, m**), green (GAL-positive: **b, f, j, n**) and blue (PGP 9.5-positive: **c, g, k, o**) fluorescent channels. Microphotographs **d, h, l** and **p** showing the overposition of all three channels simultaneously. A single CASP3/PGP 9.5-positive neuron containing GAL in cancer-unaffected areas of the human stomach wall (**a**–**d**). In the cancer-affected stomach wall numerous neurons contained CASP3/PGP9.5 (that sporadically were immunoreactive also for GAL; **e**–**h**). In the macroscopically-unchanged and tumor-adjacent part of the stomach wall a single immunoreactive for CASP8/PGP 9.5 neuron (GAL-negative, **i**–**l**, **m**–**p**; respectively). Scale bar = 50 µm. (Color figure online)

Immunofluorescent staining showed diversified pattern of co-expression of the investigated substances in the gastric MPs. For example, the percentage of neurons immunoreactive to CASP3 was significantly higher in the plexuses located close to the tumor (Fig. 1e) compared to control region of stomach wall (Fig. 1a). Whereas the distribution pattern of neurons containing GAL was opposite in these tissues (Fig. 1f and b; respectively). In turn, the percentages of neurons positive for CASP3 simultaneously with GAL (Fig. 1d and h) and immunoreactive to PGP 9.5 were similar in both studied regions (Fig. 1c and g; Table 4).

**Table 4:** The mean percentage of neurons immunoreactive to caspase-3 or caspase-8 with galanin in relation to the total number of protein gene-product 9.5-immunoreactive cells in stomach myenteric plexuses. Data were pooled and presented as the mean (range) of ten patients

Neuronal subpopulation with studied substances	Myenteric plexuses		p
	Distally from tumor	Close to tumor invasion	
Co-localization of caspase-3 with galanin [%]			
CASP3+/GAL–/PGP 9.5+	3.2 ± 1.8	8.3 ± 2.1	0.0007
CASP3–/GAL+/PGP 9.5+	13.8 ± 1.9	9.0 ± 2.5	0.001
CASP3+/GAL+/PGP 9.5+	47.9 ± 4.6	42.1 ± 6.3	ns
CASP3–/GAL–/PGP 9.5+	35.9 ± 3.0	40.4 ± 6.5	ns
Co-localization of caspase-8 with galanin [%]			
CASP8+/GAL–/PGP 9.5+	32.8 ± 4.6***	20.6 ± 4.3***	0.0001
CASP8–/GAL+/PGP 9.5+	15.8 ± 3.4	22.9 ± 4.0***	0.005
CASP8+/GAL+/PGP 9.5+	22.6 ± 4.5***	18.7 ± 5.8***	ns
CASP8–/GAL–/PGP 9.5+	28.7 ± 5.6**	36.6 ± 5.1	0.002

*ns* no statistical differences were observed, *CASP3* caspase-3, *CASP8* caspase-8, *GAL* galanin, *PGP* 9.5 protein gene-product 9.5

^**,***^indicate differences (P < 0.01; P < 0.001) between the CASP3/GAL and CASP8/GAL population of neurons within myenteric plexuses

#### Caspase-8 immunoexpression

Triple-immunofluorescent staining revealed significantly higher percentage of CASP8 and PGP 9.5 stained neurons in plexuses located distally from the tumor (Fig. 1i, k and l) when compared to the cancer-affected stomach wall (Fig. 1m, o and p). In turn, the frequency of neurons containing GAL was significantly higher in the MPs located closer to cancerous region (Fig. 1n) than in the tissue distally from it (Fig. 1j). Meanwhile the neurons from MPs containing CASP8 simultaneously with GAL showed similar percentages in both studies regions (Fig. 1l and p; Table 4).

#### Comparison of caspase-3 and caspase-8 immunoexpression

The percentage of immunoreactive for CASP3 and CASP8 neurons was different in the region close to tumor invasion compared to the region distally from it. For example, tissues located distally from, as well as close to the tumor invasion have higher percentage of cells expressing CASP8 compared to CASP3. In turn caspases co-localized with GAL have given a contrary result in both samples. Moreover, percentage of GAL-immunoreactive neurons devoid of CASP8 was significantly higher only in vicinity of tumor invasion (Table 4).

## Discussion

This is the first report that provides a detailed description of both the morphology and the neuronal chemical phenotypes of MPs distally from and in vicinity of tumor invasion.

### Morphometry of myenteric plexus (MPs)

The results of the present study showed that mean size of MPs is smaller close to tumor invasion compared to the size of the MP located in the unchanged parts of stomach. These results are mostly consistent with our previous studies in patient with colorectal cancer (CRC) [5], as well as the studies on patients with diabetes [7] or severe constipation [8]. Moreover, our results also showed that the atrophy of MPs close to tumor invasion was probably associated with apoptosis (an increased expression of CASP3). Although there were some differences as well. For example, we previously reported that in the patient with CRC the mean number of neurons was lower in proximity to tumor invasion, compared to unchanged parts of the organ [5], unlike the current study where no difference was observed between the two areas. This discrepancy may be attributed to the differences in the generally lower number of neurons in the MPs observed within human stomach wall (present study) compared to the colon wall [5] and/or differences in the arrangement of these plexuses in the gastrointestinal tract [18–20].

### Chemical phenotype of neurons in the myenteric plexuses (MPs) as revealed by triple immunochemistry

In the present study, in the cancer-affected stomach wall, the percentage of MPs neurons immunoreactive to CASP3 was significantly higher when compared to unchanged area. It is difficult to explain this phenomenon, because in the available literature there is a lack of detailed data concerning the chemical coding of neurons localized in MPs. In reference to CASP3, it has only been found that in the gastric cancer, the expression of this protein was significantly lower compared to corresponding healthy tissue [21, 22]. This difference is perhaps due to the fact that present study focuses the analysis of chemical coding of neurons in the MPs but not expression of these enzymes inside gastric cancer tissue. It should be also underline that Hu et al. [23] observed that CASP3 expression was up-regulated in chronic atrophic gastritis, intestinal metaplasia and mild-moderate atypical dysplasia, while tissues with severe dysplasia presented down-regulation of this endoprotease. Moreover, this low expression of CASP3 signified an ominous prognosis in gastric cancer [24]. In addition, the present work indicates that the percentages of neurons in the MPs expressing CASP8 decreased in the cancer-affected stomach wall. The decreased expression of CASP8 is in accordance with the study of Wang et al. [22] that was performed on gastric cancer tissue and cells. This phenomenon was not observed in previous studies including patients with CRC [5].

The present results also indicated that the percentage of GAL-IR neurons (within the CASP3-IR population of neurons) in the MPs located close to the cancer-affected stomach wall was significantly lower compared to macroscopically-unchanged part of the stomach wall. There is no detailed data describing the distribution pattern of GAL-IR neurons in the MPs of cancer-affected stomach wall. In case of this peptide, it has only been reported that in the group of patients with CRC, the percentage of the neurons containing GAL was similar in the MPs located close to and distally from tumor invasion [5]. The discrepancy in the percentage of GAL-IR neurons between the present results and Kozłowska et al. [5] may be caused by the difference in histological structure between stomach and colon wall, like the distribution of GAL receptors [25], hence the different expression and reaction to GAL.

### Functional considerations

In the present study an increase in percentage of neurons containing CASP3 in the MPs might indicate that apoptosis can be triggered by the intrinsic pathway and not by the external stimuli. It is generally known that cancer invasion requires a vast amount of energy and nutrients. In this case it is possible that energy demand of a tumor is so high that surrounding plexuses suffer from nutrient deprivation, which can lead to apoptosis caused by decreased mitochondrial respiration chain activity and ATP production. This leads to the activation of LKB1-AMPK, causing the inhibition of Raptor-mTOR complex and activation of CASP3 [26]. In addition, the present study provides the first evidence that the percentage of CASP8 neurons in the MPs close to tumor invasion was significantly lower compared to unchanged part of the stomach wall. Interestingly, the decrease in CASP8 is currently investigated as a workable target for next generation of antineoplastic drugs that could increase the overall concentration of caspase-8 and possibly trigger apoptosis [27].

The present results also indicated that higher percentage of neurons expressing CASP3 together with GAL compared to CASP8 with GAL may indicate an antiapoptotic role of GAL in the course of this disease. This observation is in accordance with study conducted by Yoon et al. [28] where the inactivation of GAL, leads to gastric cancer carcinogenesis. Those results were validated by Iischi et al. [29], where substantial doses of GAL inhibited gastric cancerogenesis. Moreover, higher expression of this peptide occurs during inflammation associated with gastric ulcers [9, 30]. It is also generally known that GAL can be regarded as an immunomodulatory peptide, because it can activate neutrophils and natural killer (NK) cells towards proinflammatory cytokines. This connection between GAL and activation of NK cells might be crucial for anti-cancerous immunologic defense [31].

Present results demonstrate that the percentage of neurons containing GAL located distally from the tumor invasion was higher, while containing CASP-3 was lower when compared to cancer-affected region. It is plausible that GAL might protecting the healthy cells against CASP-3-induced apoptosis what was previously observed in the ischemic mouse brain [32], although this hypothesis needs to be verified in detail. Furthermore, the present results indicated that the high percentage of neurons containing GAL and low percentage of CASP-8 in the MPs of cancer-affected stomach wall might also reflects neuroprotective role of this peptide. This hypothesis is supported by some interesting observations provided by Li et al. [32]. Described above results proves that fluctuation in GAL and caspases frequency are not neutral and are related with each other.

In summary, this study showed morphological changes of MPs in vicinity of gastric cancer invasion. Obtained in the present study results let us suppose that observable atrophy might be caused by apoptosis but this study does not elucidate this aspect unambiguously. Further studies are needed to clarify the role of intrinsic apoptotic pathway.
